# Axial Psoriatic Disease: Clinical and Imaging Assessment of an Underdiagnosed Condition

**DOI:** 10.3390/jcm10132845

**Published:** 2021-06-27

**Authors:** Ivan Giovannini, Alen Zabotti, Carmelo Cicciò, Matteo Salgarello, Lorenzo Cereser, Salvatore De Vita, Ilaria Tinazzi

**Affiliations:** 1 Department of Medical and Biological Sciences, Institute of Rheumatology, University Hospital ‘Santa Maria della Misericordia’, 33100 Udine, Italy; i.giovannini.qwerty@gmail.com (I.G.); salvatore.devita@asufc.sanita.fvg.it (S.D.V.); 2 Departments of Diagnostic Imaging and Interventional Radiology, IRCCS Sacro Cuore Don Calabria Hospital, 27024 Negrar di Valpolicella, Italy; carmeciccio@gmail.com; 3 Nuclear Medicine, IRCCS Sacro Cuore Don Calabria Hospital, 27024 Negrar di Valpolicella, Italy; matteo.salgarello@sacrocuore.it; 4 Department of Medicine, Institute of Radiology, University of Udine, 33100 Udine, Italy; lorenzo.cereser@asufc.sanita.fvg.it; 5 Unit of Rheumatology, IRCSS Ospedale Sacro Cuore Don Calabria, 27024 Negrar di Valpolicella, Italy; ilariatinazzi@yahoo.it

**Keywords:** axial psoriatic arthritis, psoriatic arthritis, stiffness, inflammatory back pain

## Abstract

The frequent involvement of the spine and sacroiliac joint has justified the classification of psoriatic arthritis (PsA) in the Spondyloarthritis group. Even if different classification criteria have been developed for PsA and Spondyloarthritis over the years, a well-defined distinction is still difficult. Although the majority of PsA patients present peripheral involvement, the axial involvement needs to be taken into account when considering disease management. Depending on the definition used, the prevalence of axial disease may vary from 25 to 70% in patients affected by PsA. To date, no consensus definition has been reached in the literature and the definition of axial involvement in PsA has varied from isolated sacroiliitis to criteria used in ankylosing spondylitis. This article reviews the unmet needs in the clinical and radiological assessment of axial PsA, reporting the various interpretations of axial involvement, which have changed over the years. Focusing on both imaging and clinical standpoints, we reported the prevalence of clinical and radiologic features, describing the characteristics of axial disease detectable by X-rays, magnetic resonance imaging, and PET-CT, and also describing the axial symptoms and outcome measures in patients affected by axial disease.

## 1. Introduction

Psoriatic arthritis (PsA) is a common chronic and potentially debilitating inflammatory arthropathy, affecting 0.4–2% of the general population [1], and from 7 to 42% of psoriasis patients develop arthritis [2]. PsA may begin with several clinical features such as peripheral arthritis, dactylitis, enthesitis, and axial disease [3,4]; dactylitis and enthesitis in particular have a relevant role in the early identification of PsA. 

The frequent involvement of the spine and sacroiliac joint has justified its classification in the Spondyloarthritis (SpA) group [5]. The PsA prevalent phenotype is not often fixed at the onset of the disease but may vary during the early phase of its development [6]. The majority of PsA patients present peripheral involvement [7], which has been largely studied over the years, but a widely accepted definition of axial involvement remains problematic. There are various ways to define axial disease in PsA, such as alone or in combination with peripheral disease, according to inflammatory axial symptoms based on radiographic signs (radiographic sacroiliitis, other radiographic signs of spondylitis) [8].

Few studies compare axial PsA to ankylosing spondylitis (AS) and axial SpA [9], and similar imaging and clinical scoring methods are currently used in the management of axial manifestations.

The axial involvement in PsA was described more than 50 years ago, with the detection of changes in the sacroiliac joints, such as erosion and sclerosis [10]. The definition of axial involvement has changed over the years, from unilateral sacroiliitis to similarities with AS [11]. The prevalence of axial disease depends on the duration of the disease; axial PsA ranges from 5–28% in early PsA, and 25–70% in established PsA [11,12,13], suggesting that axial disease develops progressively during the course of PsA. 

In the Toronto PsA cohort, 15% of PsA patients without axial involvement at baseline are estimated to develop axial PsA during a follow-up period of 10 years [7]; the risk factors associated with axial PsA onset were HLA B27, nail dystrophy, high number of radiographically damaged joints, periostitis and elevated erythrocyte sedimentation rate (ESR) [7]. 

Cervical axial PsA occurs in 35–75% of cases [14,15,16], more frequently in patients with longstanding and severe disease [15]. Two types of radiological lesions are reported in the cervical spine: upper and lower cervical involvement. The first is often characterized by C1-C2 arthritis, such as erosion of the odontoid and atlanto-axial instability, potentially leading to spinal cord compression [17]. As reported by Salvarani et al. [14], the frequency of atlanto-axial subluxation (defined as an atlanto-dens interval of more than 4–5 mm) and odontoid erosion in an Italian cohort of PsA patients were 12% and 23%, respectively. The lower cervical spine involvement may be characterized by syndesmophytes, osteitis of the inter-apophyseal joints and ossification of the anterior longitudinal ligament [16,17], rarely complicated with neurological deficit. 

The definition of sacroiliitis is still debatable. Battistone et al. [18] defined the prevalence of radiographic evidence of sacroiliitis in a PsA cohort by the radiographic features derived from AS, such as the New York criteria [19]. The average duration of the disease was 12 years and, in the 202 patients enrolled, the prevalence of radiographic evidence of sacroiliitis (grade 2 or higher) was 78% with 71% of these presenting grade 3 disease [18]. At present, there is not a widely accepted definition of axial PsA [20]. Overall, asymptomatic sacroiliitis evaluated by conventional radiography has been reported in about one third of PsA patients [21,22], and the inflammation in the axis may also cause inflammatory back pain (IBP), stiffness and radiographic changes, such as sacroiliitis, spondylitis and syndesmophytes [23,24]. 

## 2. Symptoms, Changes and Outcome Measure

At present there is no clear distinction between axial involvement in PsA and the other spondyloarthritis (SpA). The current classification criteria for SpA (ASAS) [25] and PsA (CASPAR) [26] overlap. 

PsA has a polymorphic clinical phenotype, the majority of patients present peripheral disease as an early onset symptom and only the minority are diagnosed as having exclusive axial involvement [8]. Often, axial involvement is detected by a radiographic assessment during the course of the disease. Disease activity indexes are also essentially based on peripheral symptoms, such as arthritis, dactylitis and enthesitis [21]. 

IBP is the most typical symptom associated with axial disease, as well as AS [27], and is reported by 15–19% of the PsA patients [28,29,30]. In a recent study, Yap et al., evaluated the performance of various IBP criteria (such as Calin, Berlin and ASAS criteria) in the detection of axial PsA, reporting a low sensitivity and a specificity between 73 and 82% of the 3 criteria in a cohort of patients presenting axial disease on radiographic or MRI imaging [30,31]. Stiffness and cervical pain are also common symptoms in PsA; in fact, in a cohort of established PsA, these symptoms were reported in 24% of the patients, and 41% presented radiographic involvement in the cervical spine, leaving 17% of patients with radiographic disease but no symptoms [15]. 

These data support the idea that clinical symptoms are not accurate predictors of cervical spine involvement, therefore erosive and progressive PsA might be screened for atlanto-axial instability, a potentially serious complication. 

Recently, the CORRONA registry database included data from 2330 PsA patients from 25 states in the United States. A sub-analysis about axial-PsA involvement was performed in the CORRONA registry among patients enrolled between March 2013 and March 2016. Axial involvement was defined as physician-reported spinal involvement at enrolment and/or when imaging showed sacroiliitis; patients without axial symptoms were defined as patients with no axial involvement. According to this definition, axial PsA was described in 12.5% of the 1530 PsA patients enrolled [32]. Patients with axial disease tend to be younger and more prone to present a previous use of biologic treatment compared to patients without axial involvement [32]. Mease et al., also highlighted that axial PsA occurred in patients with severe arthritis, enthesitis, and severe skin and nail psoriasis [32]. Patients with axial PsA also reported higher pain and fatigue, measured with a visual analogue scale (VAS) and performed worse in the patient-reported outcomes (PROs), such as the Health Assessment Questionnaire (HAQ), work production and activity impairment (WPAI) and morning stiffness. Axial patients are more prone to depression and discomfort at baseline than non-axial patients, as reported using EQ-5D-3L [32]. 

To date, there is no specific and shared tool to measure clinical axial involvement in PsA. The more usual scoring measures have been borrowed from AS, such as the Bath Ankylosing Spondylitis Disease Activity Index (BASDAI), the Ankylosing Spondylitis Disease Activity Score (ASDAS), and the Bath Ankylosing Spondylitis Functional Index (BASFI). The INSPIRE study (International Spondylarthritis Interobserver Reliability Exercise) tested whether the axial measures used in AS were also reproducible for axial PsA [33,34]. They reported the best reliability among experts to discriminate axial PsA using BASFI, modified Schober test, lateral lumbar flexion, finger to floor distance, and the BASDAI. BASDAI, however, is also influenced by peripheral joint disease. 

## 3. Imaging Changes

### 3.1. Radiographic Changes 

Radiographic axial PsA was first reported in 1961 [10], when it was recognized that sacroiliac changes in PsA (erosions, sclerosis, and ankylosis) are more frequent compared to RA. 

Nowadays, the modified New York criteria [19] are used to assess radiographic spinal changes. However, these changes may develop after years and may be confused with degenerative changes [35], as most patients demonstrate onset of the disease in the 4th and 5th decade. As previously reported, the prevalence of axial disease in PsA patients varies with the duration of the disease. In fact, radiographic changes at baseline range from 7 to 17% [36].

Although there are no clear discriminative radiographic scores differentiating axial PsA and AS, published data suggest that axial disease in AS has a worse clinical impairment compared to axial PsA. Radiographic sacroiliitis in PsA was reported more frequently unilateral and less severe than in AS [37,38]. There is usually a larger volume of syndesmophytes in PsA that do not follow the anterior longitudinal ligament course, such as in AS where syndesmophytes appear in consecutive vertebrae [23]. As described by Salvarani et al. in a northern Italian cohort, longstanding PsA patients present signs of radiographic cervical involvement in 70–75% of cases [14]. The high prevalence of radiographic cervical spine involvement was successively reported in 41% by Queiro et al. in 2002. In this Spanish cohort, 58.5% of patients complained of cervical pain and stiffness, whereas 41.5% presented radiologic changes without symptoms [15]. 

A specific radiographic scoring system for axial PsA was developed by Lubrano et al., called PASRI (Psoriatic Arthritis Spondylitis Radiology Index) [39]. This scoring system combines features from the AS-based radiological index, such as BASRI (Bath AS Radiology Index) and mSASSS (modified Stoke AS Scoring System). 

Ibrahim et al. [40] reported that PASRI presented a moderate sensitivity (0.52) but high specificity (0.74) for an increase in scores to detect true change. They also evaluated BASRI-spine and mSASSS, describing that the sensitivity to measure change, as judged by the external expert, was only approximately 50%. PASRI has the advantage over BASRI and mSASSS to detect posterior axial involvement, thus, in assessing the sacroiliac joints and the cervical and lumbar spine, PASRI might extend the radiological assessment of axial PsA to also consider the facet joints of the spine.

### 3.2. Magnetic Resonance Imaging Changes 

Magnetic Resonance Imaging (MRI) allows a high-resolution visualization of the structures involved in arthritis. The knowledge of MRI in PsA derives from studies of SpA patients, including limited numbers of PsA patients [41]. Signs of inflammation, although non-specific for PsA, may be detected by MRI, such as synovitis, tenosynovitis and bone marrow oedema [42]. In patients with SpA, including axial PsA, MRI is the reference standard technique to identify non-radiographic axial inflammation, and active axial involvement. Few studies have evaluated the assessment of spine and sacroiliac involvement in axial PsA using MRI [43], but overall MRI findings in axial PsA were compared to those in AS. MRI is a sensitive diagnostic technique to detect sacroiliitis and spondylitis early changes. Williamson et al., described features of sacroiliitis in 38% of the PsA patients and abnormal MRI scans were associated with restricted spinal movements [44]. Usually, clinical findings in PsA are not strongly associated with MRI sacroiliitis, as reported in a 2017 study on 135 axial MRI images [45], but, in adjunct to clinical examination, may influence the treatment change in symptomatic patients in 56% of the cases. A relationship between the severity and extent of axial inflammation and HLA-B*27 genotype was highlighted in PsA, comparable with AS patients [46]. This supports the idea that HLA-B*27 positive patients are more prone to present more severe axial bone marrow oedema. 

AxSpA MRI lesions of the spine mainly consist of bone marrow oedema/osteitis related to inflammation at the intervertebral disc’s enthesis. Typical locations of inflammation are the anterior and posterior vertebral body corners. The Assessment in Spondylarthritis international Society–Outcome Measures in Rheumatology (ASAS/OMERACT) MRI working group suggested that the presence of anterior/posterior spondylitis in three or more sites is highly suggestive of axial SpA, giving a minor role to other sites of inflammatory lesions [47]. More recently, the Canada–Denmark (CANDEN) MRI working group developed and validated a comprehensive system defining and scoring both inflammatory and structural lesions of the spine in patients with axSpA [48,49,50]. Taking into account both body and posterior elements of each vertebra, the CANDEN scoring system scores the entire spine, thus providing detailed monitoring of changes over time in patients with known axSpA [49,50]. It has been supposed that posterior elements involvement (e.g., costovertebral and facet joints) may have greater specificity for axSpA than vertebral body locations (i.e., corner and non-corner lesions) [51]. Moreover, vertebral bodies are also often affected by degenerative disc disease in healthy subjects. The application of the CANDEN scoring system may help understand the evolution of inflammatory and structural lesions at different vertebral locations and how different drugs may influence them in the whole spine, but it is not easy to apply in clinical practice. 

The CANDEN scoring system requires a dedicated MRI protocol, with high-field scanners (i.e., equal, or superior to 1 Tesla) using 4-mm-thickness T1-weighted turbo spin-echo and short tau inversion recovery (STIR) sequences on the sagittal plane. Separate cervical, thoracic, and lumbar spine acquisitions should compose the examination. Of note, sagittal slices must include the spinal canal (i.e., central sagittal slices) and the pedicles (i.e., lateral sagittal slices) [51]. Lateral sagittal slices allow the evaluation of posterior elements of the spine (i.e., facet joints, transverse processes, ribs, and soft tissues) [51]. All that being considered, a state-of-the-art MRI examination of the spine in patients with known or suspected axSpA must not leave out technical care, as well as the radiologist’s accurate interpretation and clinically relevant reporting.

Whole-body MRI is a useful screening tool to detect bone lesions in multiple myeloma as it can provide, in less than an hour, a complete assessment of the whole body [52]. It also allows the simultaneous evaluation of PsA domains such as enthesitis, dactylitis and axial involvement [52]. MRI is linked to a more sensitive assessment of inflammatory and structural lesions, compared with clinical outcomes and X-rays [53]. According to the whole-body MRI studies of Poggenborg et al., bone marrow oedema is more frequent in PsA and SpA than in healthy subjects, and this technique may show the whole distribution of inflammatory and structural lesions [54,55]. This technique was further examined by Mager et al., as a diagnostic tool for early spondylarthritis detection [56] but it needs more research to be used in clinical practice, including the image acquisition. 

### 3.3. FNa 18 PET CT 

**Figure 1:**
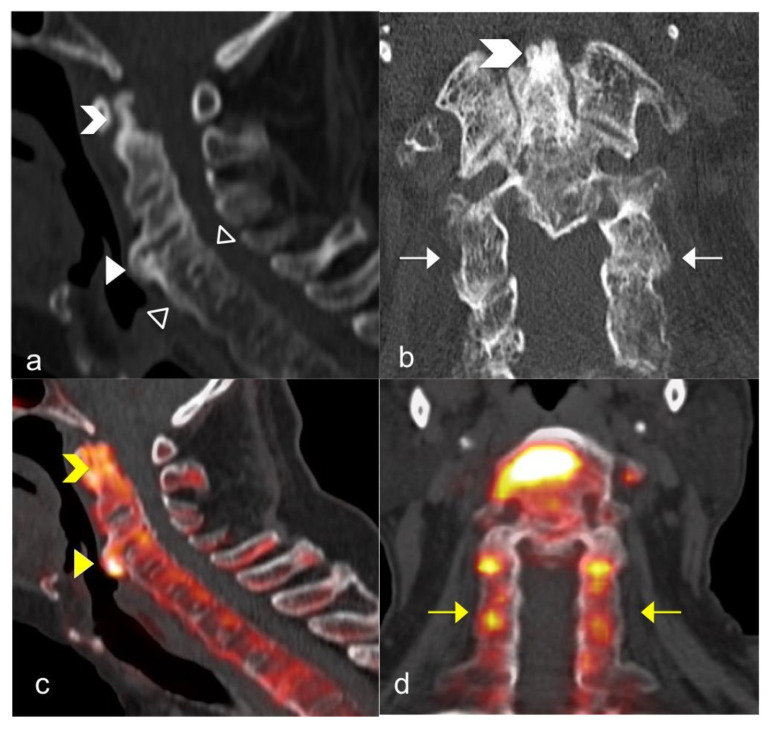
A 66-year-old man with axial PsA. The CT image on the sagittal plane (**a**) shows extensive new bone formation with bridging-syndesmophytes along the anterior and posterior corner (empty white arrowheads) also with bridging-osteophytes between C3-C4 level (white arrowhead); CT images on the coronal plane (**b**) show extensive structural damage with bony erosion and sclerosis of dens (white chevron) also with multilevel ankylosis of facet joints (white arrows). Sodium 18F-Fluoride PET-CT of the cervical spine on the sagittal (**c**) and coronal plane (**d**) shows an increase in tracer uptake at the dens (yellow chevron), at the bridging-osteophytes between C3-C4 level (yellow-arrowhead) and along the facet joints (yellow-arrows).

**Figure 2:**
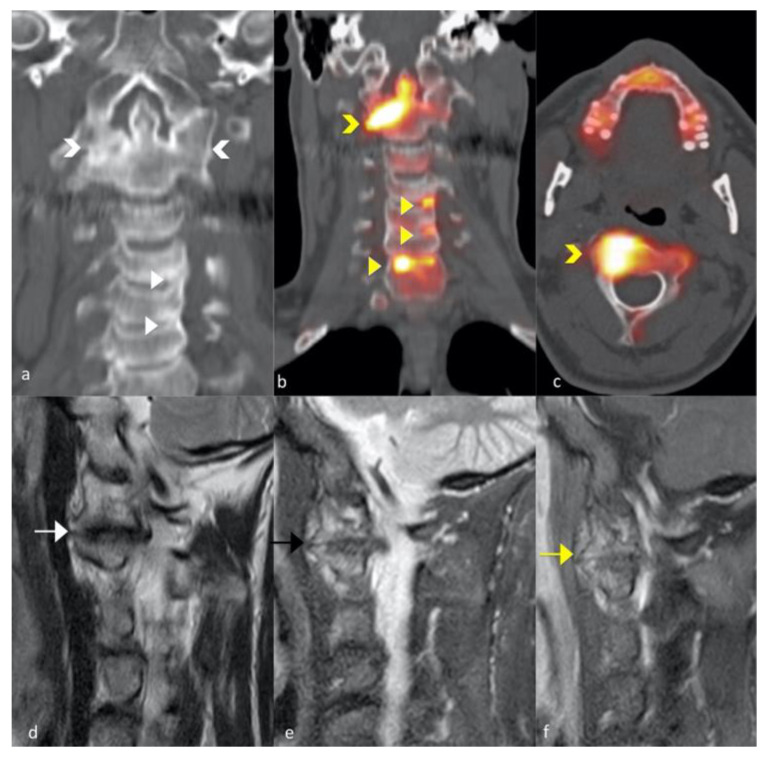
A 56-year-old man with axial PsA. CT coronal image of the cervical spine (**a**) shows the fusion of both atlanto-axial articulations; huge horizontal oriented osteophytes with extensive sclerosis of the articular cortical bone was observed at the right atlanto-axial articulation. Sodium 18F-Fluoride PET-CT of the cervical spine on the coronal (**b**), axial (**c**), and saggital plane (**d**) shows the increase in tracer uptake at the dens in extension to right lateral mass (yellow chevron); focal areas with increase in tracer uptake were observed at the antero-lateral corners on the left side of C3-C4 and C4-C5 levels and on the right antero-lateral corner of the vertebral plate of C7 (yellow-arrow-heads); the corresponding CT image (**a**; arrowheads) shows signs of structural damages with bone sclerosis also with small cortical erosions. Sagittal T2-weighted and short tau inversion recovery (STIR) T2-weighted TSE images (**d**,**e**) show signs of structural damages at the right atlanto-axial articulation with bone formation and bone oedema with the involvement of periarticular soft tissues (arrows); sagittal fat-suppressed gadolinium-enhanced T1-weighted FS TSE (**f**), show contrast enhancement of articular space, also involving cortical bone and periarticular soft tissue, in keeping with active synovitis (yellow arrow).

Since the syndesmophyte formation is not clearly identifiable on MRI, new techniques dedicated to bone remodelling, such as PET-CT, may play a role [57]. In recent years, the PET diagnostic potential has been considered in both infectious and inflammatory diseases, such as polymyalgia rheumatica, vasculitis, and rheumatoid arthritis [58]. 18 F sodium fluoride (18NaF) is an imaging tracer of calcium metabolic activity of bone structures and soft tissue (Figure 1 and Figure 2). This tracer is confined to detect bone metastasis, but various studies have revealed its role in predicting progression in osteoporosis, osteoarthritis, rheumatoid arthritis, and ankylosing spondylitis. The visualization of physiological change makes FNa18 PET potentially suitable for early detection of SA spine disease, even before anatomical changes occur. Recently, Son et al. [59] evidenced the accuracy of 18NaF PET to detect AS typical lesions such as enthesopathy, sacroiliitis, and syndesmophytes, suggesting a role for this technique to diagnose AS and to also monitor therapeutic effects. 

## 4. Conclusions

Spine involvement in PsA is a frequent and underdiagnosed feature, particularly in the early phase of the disease [60]. Typically, PsA onset occurs later compared to AS, and thus, spine involvement may also be associated with degenerative changes. In axial disease, sclerosis and osteophytes may be present both in inflammatory and degenerative disease, and the correct attribution is complicated. As previously reported, the prevalence of axial involvement in PsA varies between 5% and 78% [11,12]. This heterogeneity is related not only to the duration of the disease in the considered cohort, but also to the definition and inclusion criteria used by the different authors. Some studies required radiographic evidence of sacroiliac disease fulfilling the modified New York criteria [18,37,61], other studies prefer to concentrate on symptoms, such as inflammatory back pain [7,32]. This lack of definition and radiological assessment does not allow for the accurate determination of the incidence and the degree of progression of the disease. Furthermore, cervical spine involvement in PsA is not always associated with low back pain and arises more often with stiffness, limitation of flexion and rotation than with pain [15]. 

To date, spine involvement in PsA is not clearly defined, neither from a clinical nor radiological standpoint, making it difficult to ensure a precise diagnosis and follow-up. In clinical practice, but also in clinical trials, spine assessment using imaging is not frequent, except for cases in which the spine involvement is predominant or drives the therapeutic decision. Thus, the presence of radiological axial involvement in patients who do not complain about axial symptoms may modify the therapeutic approach according to EULAR recommendations [62]. Furthermore, in case of axial pain in PsA, conventional radiography and MRI are diagnostic tools useful for the differential diagnosis and for the identification of imaging lesions specific for PsA. 

The clinical assessment of axial involvement in PsA relies on subjective or patient-reported measures of disease activity already in use in AS, such as ASDAS, BASDAI, BASFI. An important limitation is that many of the items are redundant [63]. 

The therapeutic approach in axial PsA is currently of great interest. To date, according to the EULAR recommendations [62], if axial disease predominates and presents insufficient response to non-steroidal anti-inflammatory drugs, treatment with TNF inhibitor or IL-17A inhibitor should be considered. Furthermore, IL12/23 inhibitor, such as ustekinumab and IL23 inhibitor, such as risankizumab, did not prove clinical efficacy in axial disease [64,65,66]. Thus, more studies are needed to clarify why a drug that inhibits IL23, which acts upstream from IL17, presents no clear clinical efficacy in axial disease, whilst having a good effect in psoriasis [67]. Preliminary results are also available on JAK inhibition in axial disease. Among JAK inhibitors, filgotinib efficacy and safety was recently described in the TORTUGA trial [68], and previously also tofacitinib and upadacitinib showed preliminary usefulness in axial disease [69,70], leading the way to assess the performance of JAK inhibitors in PsA with axial involvement [71].

New imaging tools are needed to identify spinal PsA involvement for drive treatment strategy and to test the efficacy of new drugs. These tools, using MRI for early lesions or X-rays for established ones, should be easy and feasible as AS clinical tools, such as BASDAI, ASDAS and BASFI, do not seem specific enough. 

## 5. Research Agenda

There is an increasing need to correctly define and identify axial PsA without using AS clinical and imaging tools. Nowadays, there is no specific clinical assessment for axial PsA; further research may consider a new tool based on PsA patients’ axial symptoms.

A modified index for radiologic assessment of axial involvement in psoriatic arthritis (PASRI) was proposed in 2008 [39]: it included existing radiologic features of AS, with the addition of scores only for cervical facet joints, because the assessment of lumbar facet joints on plain AP and lateral radiographs was considered problematic. This score did not include the dorsal spine and was not used in many clinical trials. 

The gold standard technique to identify early spine involvement and inflammatory alterations on posterior elements is MRI, but information on syndesmophytes morphology and sclerosis could be missed. A new integrated spine PsA assessment using X-rays and MRI should be tested to identify the underdiagnosed progressive axial disease. The aim may be to screen patients correctly at the onset of the disease. Further research in Nuclear Medicine imaging using a calcium metabolism tracer is needed to test the role of NaF18 in axial PsA identification and quantify its metabolic activity. Since data from PET imaging are quantitative, this technique adds a major dimension to assess disease activity and is a promising strategy to identify disease progressors. 

Prospective and longitudinal cohorts are needed to allow the characterization of axial involvement in PsA and to aim a consensus definition of axial PsA. Once a definition is formulated, an enhancement in the assessment tools and management strategies is desirable. 

## Data Availability

Data sharing not applicable.
